# MiR-185 targets POT1 to induce telomere dysfunction and cellular senescence

**DOI:** 10.18632/aging.103541

**Published:** 2020-07-18

**Authors:** Tingting Li, Zhenhua Luo, Song Lin, Chujun Li, Shenkun Dai, Haoli Wang, Junjiu Huang, Wenbin Ma, Zhou Songyang, Yan Huang

**Affiliations:** 1 MOE Key Laboratory of Gene Function and Regulation, Institute of Healthy Aging Research, School of Life Sciences, Sun Yat-sen University, Guangzhou, China; 2 Institute of Precision Medicine, The First Affiliated Hospital, Sun Yat-Sen University, Guangzhou, China; 3 Guangzhou First People’s Hospital, School of Medicine, South China University of Technology, Guangzhou, China

**Keywords:** cellular senescence, protection of telomere 1, miR-185, telomere dysfunction, aging

## Abstract

Protection of telomere 1 (POT1), the telomeric single-stranded DNA (ssDNA)-binding protein in the shelterin complex, has been implicated in the DNA damage response, tumorigenesis and aging. Telomere dysfunction induced by telomere deprotection could accelerate cellular senescence in primary human cells. While previous work demonstrated the biological mechanism of POT1 in aging and cancer, how POT1 is posttranscriptionally regulated remains largely unknown. To better understand the POT1 regulatory axis, we performed bioinformatic prediction, and selected candidates were further confirmed by dual-luciferase reporter assay. Collectively, our results revealed that miR-185 can significantly reduce POT1 mRNA and protein levels by directly targeting the POT1 3’-untranslated region (3’-UTR). Overexpression of miR-185 increased telomere dysfunction-induced foci (TIF) signals in both cancer cells and primary human fibroblasts. Elevated miR-185 led to telomere elongation in the telomerase-positive cell line HTC75, which was phenotypically consistent with POT1 knocking down. Moreover, miR-185 accelerated the replicative senescence process in primary human fibroblasts in a POT1-dependent manner. Interestingly, increased serum miR-185 could represent a potential aging-related biomarker. Taken together, our findings reveal miR-185 as a novel aging-related miRNA that targets POT1 and provide insight into the telomere and senescence regulatory network at both the intracellular and extracellular levels.

## INTRODUCTION

Telomere is a specialized protein-DNA structure that caps the linear chromosome ends. Telomeric DNA components consists of double-stranded and single-stranded TTAGGG tandem repeats. In mammals, the double-strand region is several thousand base pairs in length, while the single-strand region is several hundred nucleotides in length. The telomeric protein complex, known as shelterin or telosome, is composed of six core proteins: the double-stranded repeat binding protein TRF1 and TRF2; the single-stranded repeat binding protein, protection of telomere 1 (POT1)/TPP1 heterodimer; the central bridge protein TIN2, which connects the double and single strands; and the TRF2-binding protein RAP1. Coordination between telomere replication and capping during the cell cycle ensures telomere maintenance, further guaranteeing genome stability [1, 2].

In normal human somatic cells, hardly any telomerase activity can be detected. Due to a replication problem, telomere attrition involves approximately 100 base pairs per cell division cycle. With the gradual erosion of telomeres, somatic cells eventually undergo replicative senescence, which may be a protective mechanism by which cells prevent tumorigenesis [3]. Defective telomere replication, inappropriate capping, or progressive cell divisions that reach the “Hayflick limit” can disrupt the homeostasis of telomeres. Phenotypes such as cell cycle arrest, telomere dysfunction-induced foci (TIF) formation, telomere end-to-end fusion, and telomere shortening can ultimately trigger a series of telomere-relevant diseases, including premature aging syndromes such as bone marrow failure and dyskeratosis congenital (DC) as well as cancer. Collectively, mutations in several genes, including TERT, TERC, TINF2 and ACD (TPP1), were found to be related to DC [4].

As one of the six core components of shelterin, POT1 is a unique single-stranded DNA (ssDNA)-binding protein that forms a heterodimer with TPP1, which is loaded onto the very end of the telomeric 3’ overhang by TIN2 tethering. Telomere capping by POT1 efficiently prevents telomeric 3’ overhangs from being exposed as DNA damage sites, thus repressing the activation of ataxia telangiectasia and Rad3-related kinase (ATR) signaling pathway. POT1 antagonizes RPA binding to telomeric ssDNA. POT1 depletion causes RPA to aberrantly accumulate at telomeres and activates the ATR-mediated checkpoint response. Inhibition of POT1 could activate ATR-dependent DNA damage signaling and induce telomere fragility, replication fork stalling, and telomere elongation [5, 6]. POT1 is a key protein linking senescence and tumorigenesis. POT1 deficiency in both mouse (Pot1a) and primary human cells can lead to premature senescence [6–9]. Telomere length is considered as the “molecular clock” of senescence. Increasing evidence has demonstrated that POT1 negatively modulates telomere length. Successive knockdown of POT1 or overexpression of the 5’-OB fold deletion mutant form of POT1 led to telomere elongation in the telomerase-positive cell line HTC75, a cell line derived from HT1080 fibrosarcoma cells [10].

POT1 is crucial for stem cell function. Previously we found that the POT1 homolog in the flatworm is required for homeostasis and regeneration [11]. Interestingly, the expression of Pot1a in hematopoietic stem cells (HSCs) was shown to decrease significantly with age in vitro, and Pot1a KO reduced the long-term repopulation (LTR) activity of HSCs [12]. Inactivation of POT1a in a p53-null context exacerbated the initiation of malignancy in mice; consistently, POT1 mutations were shown to cause predispositon to several types of malignant tumors, including cutaneous malignant melanoma, chronic lymphocytic leukemia (CLL), glioma and cardiac angiosarcoma [13–18]. Decreased POT1 gene expression is also associated with short telomeres in patients with severe aplastic anemia [19]. Aging is a systemic phenomenon regulated by multiple pathways. Cellular senescence acts as a barrier to cancer progression. In eukaryotes, microRNAs (miRNAs) regulate gene expression during many cellular processes. To date, senescence-associated miRNAs (SA-miRNAs) have been identified to contribute to tumor suppression. For example, miR-34a is induced during the aging process and increases age-related and myocardial infarction-induced cardiomyocyte cell death [20]. MiR-217 targets silent information regulator 1 (SIRT 1) and modulates endothelial cell senescence [21]. MiR-22 induces the senescence program in cancer cells and acts as a tumor suppressor [22]. Recent findings also indicated crosstalks between telomeric proteins and miRNAs. Previously, we performed high-throughput screening and found that miR-23a targets TRF2 to induce telomere dysfunction and cell senescence [23]. However, the SA-miRNAs that target other shelterin components remain largely unknown.

POT1 serves as a central platform that modulates players from diverse signaling pathways, linking senescence and tumorigenesis. Although the function of POT1 is well known, how POT1 is regulated, especially in the post-transcriptional level, remains largely unclear. Here, we hypothesized that POT1 can be regulated by miRNAs. We predicted candidate miRNAs with bioinformatic tools and further validated them by luciferase assay and qRT-PCR. We found that overexpression of miR-185 induces telomere dysfunction in both cancer cells and primary human somatic cells. Moreover, miR-185 accelerated the senescence process in primary human cells through downregulating POT1. We also provide evidence that miR-185 levels in sera are correlated with human age. Our findings demonstrate miR-185 as a novel aging related miRNA and provide insight into anti-aging drug development.

## RESULTS

### Bioinformatic analysis and a dual-luciferase reporter assay revealed that miR-185 can target POT1 at its 3’-UTR

**Figure 1:**
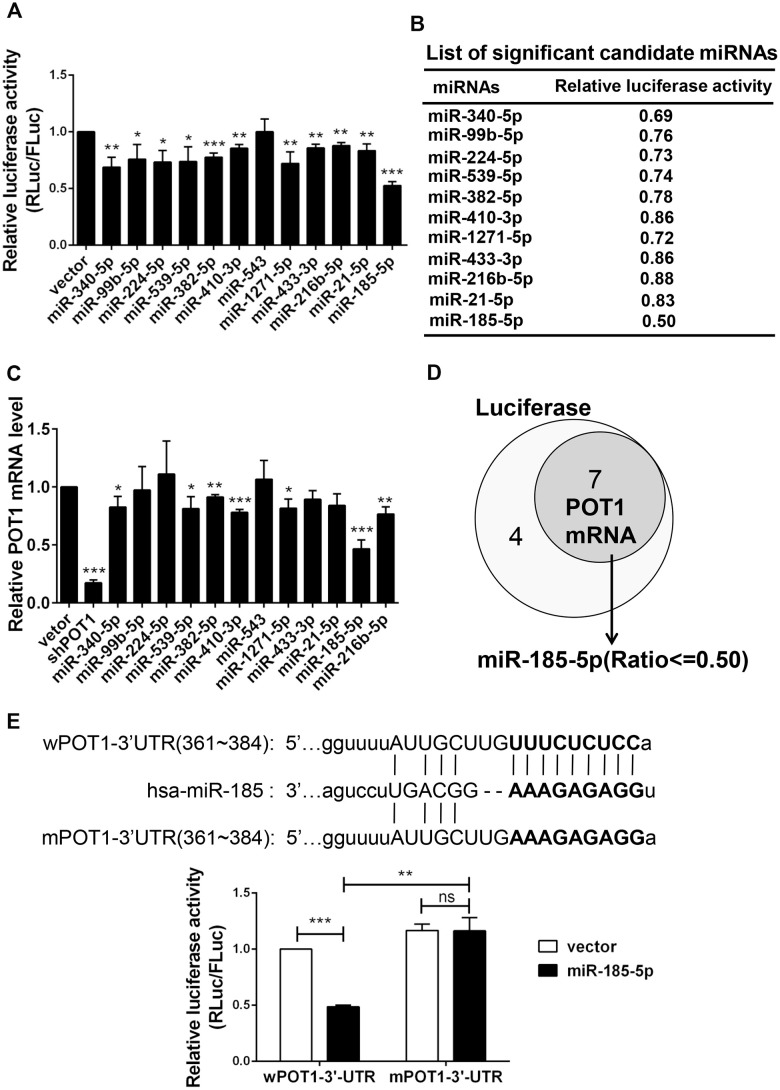
**Identification of miR-185 targeting the 3’-UTR of shelterin POT1.** (**A**) Transient dual luciferase reporter assay of HEK293T cells following expressing each candidate miRNA and the POT1 3’-UTR. P values were determined by Student’s t-test. *P<0.05, **P<0.01, ***P<0.001. (**B**) List of significant candidate miRNAs and the relative luciferase activities of cells from (**A**). (**C**) Relative POT1 mRNA level after overexpression of each candidate miRNA in HTC75 cells. P values were determined by Student’s t-test. **P<0.01, ***P<0.001. (**D**) Venn diagram of significant candidate miRNAs that downregulate either POT1 3’-UTR luciferase activity or endogenous POT1 mRNA levels. (**E**) The predicted seed region of the miR-185 target sites was mutated (in bold). The ability of miR-185 to target the seed regions of the wild-type (wPOT1) vs. mutant (mPOT1) 3’-UTR was determined by dual luciferase assay. P values were determined by Student’s t-test. **P<0.01, ***P<0.001.

To identify candidate miRNAs that target telomeric proteins, we used the online prediction tool ENCORI (The Encyclopedia of RNA Interactomes) (http://starbase.sysu.edu.cn/index.php) to predict miRNAs targeting shelterin proteins. ENCORI is an open-source platform focused on miRNA-target interactions [24, 25]. We previously identified that miR-23a targets TRF2 to induce cell senescence [23]. As deficiency in POT1 can also induce cell senescence, we focused on miRNAs targeting POT1. The results identified 16 miRNA candidates, all of which were predicted by the miRanda program (Supplementary Table 1) [26]. Notably, all predicted miRNA targeting regions are located in the POT1 3’-untranslated region (3’-UTR). We selected 12 miRNAs from an established miRNA expression library and performed a dual-luciferase reporter assay [27]. The result showed that 11 of these 12 miRNAs decrease POT1 3’-UTR luciferase activity in HEK293T cells by transient co-transfection (Figure 1A, 1B). We then stably transfected these 12 miRNAs into HTC75 cells and confirmed that 7 of them could decrease POT1 mRNA levels (Figure 1C). MiR-185 was the only candidate whose overexpression could downregulate both the reporter luciferase activity and the endogenous POT1 mRNA levels by over 50 %, so we then focused on miR-185 for further studies (Figure 1D).

By using the miRNA prediction tool starBase v2.0, we derived a putative miR-185 target site within the POT1 3’-UTR (361-384), which is conserved in only several primates including human (Supplementary Figure 1). To determine whether this putative site is targeted by miR-185, the seed region was mutated and named as mPOT1-3’-UTR (Figure 1E, upper panel). By using dual luciferase reporter assay, we compared the effects of miR-185 overexpression on wild-type POT1 (wPOT1-3’-UTR) or mutant POT1 (mPOT1-3’-UTR) luciferase activity. The wild-type POT1-3’-UTR luciferase activity was significantly reduced by ~50% upon miR-185 overexpression, while the mutant activity remained unchanged, indicating that the putative miR-185 seed region is indeed crucial for the interaction between miR-185 and POT1-3’-UTR (Figure 1E, lower panel). These results indicated that the 3’-UTR of the shelterin POT1 is specifically targeted by miR-185.

### MiR-185 induced telomere deprotection by downregulating POT1 protein levels in cancer cells

**Figure 2:**
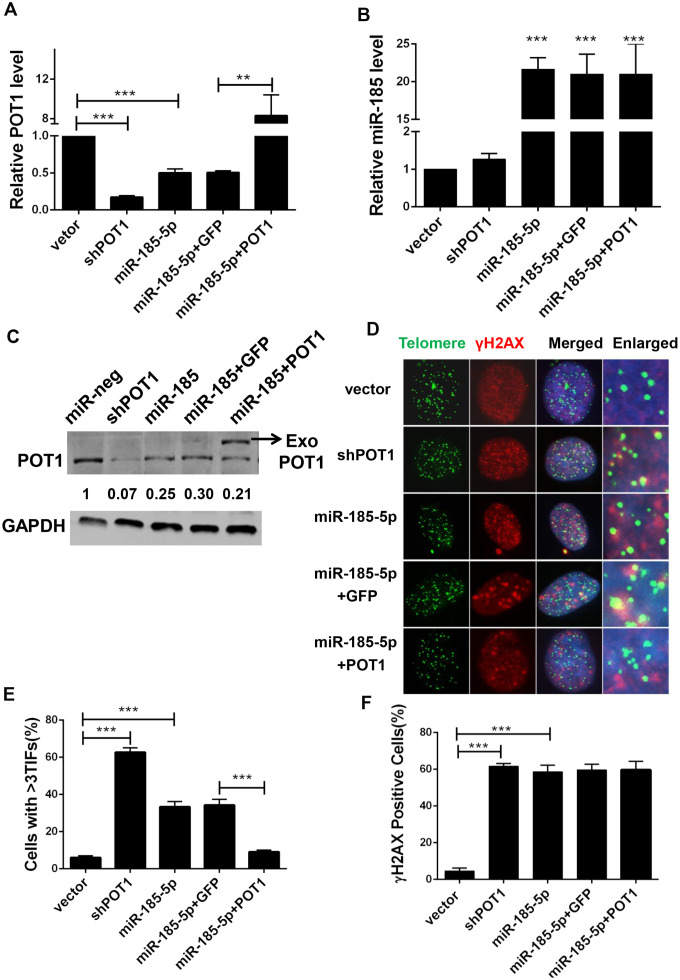
**MiR-185 induces DNA damage foci at dysfunctional telomeres by decreasing POT1 protein level in cancer cells.** (**A**) POT1 mRNA levels in POT1 knockdown, miR-185 overexpressing and POT1 rescue HTC75 cells. (**B**) miR-185 levels in POT1 knockdown, miR-185 overexpression and POT1 rescuing HTC75 cells. (**C**) POT1 protein level in POT1 knocking-down, miR-185 overexpression and POT1 rescue HTC75 cells. The arrowhead indicates exogenously overexpressed POT1. GAPDH was blotted as a loading control. (**D**) Immunofluorescence and fluorescence in situ hybridization (IF-FISH) were performed in POT1 knockdown, miR-185 overexpression and POT1 rescue HTC75 cells with the indicated γ-H2AX antibody and the TTAGGG telomere probe. Nuclei were stained with Hoechst 33342. (**E**) Quantification of percentage of cells in (**D**) with more than 3 TIFs. Error bars indicate standard deviations (n=3). P values were determined by Student’s t-test. ***P<0.001. (**F**) Quantification of the percentage of cells with signal indicating total DNA damage (γ-H2AX positive cells) in (**D**).

Given the crucial role of POT1 in telomere protection, we speculated that miR-185 overexpression would inhibit POT1 expression and disrupt telomere function. We stably transfected miR-185 into HTC75 cells and observed a decrease in POT1 mRNA levels (Figure 2A, 2B). We also rescued POT1 expression with a miR-185-insensitive POT1 expression plasmid (Figure 2A, 2B). Consistent with the change in mRNA levels, POT1 protein levels were also decreased after miR-185 overexpression and could be rescued after the reintroduction of POT1 (Figure 2C). As POT1 depletion will activate ATR signaling and induce TIF signals in cells, we next examined the effect of miR-185 on telomere deprotection. As a positive control, POT1 knockdown caused a significant increase in TIF-positive cells (Figure 2D, 2E). Consistently, overexpression of miR-185 also led to a significant increase in TIF-positive cells (Figure 2E). Furthermore, POT1 reintroduction rescued TIF signals in miR-185 overexpressing cells, suggesting that the specific mechanism by which miR-185 induces telomere dysfunction occurs via POT1 (Figure 2D, 2E). Noticeably, both the overexpression of miR-185 and the knockdown of POT1 increased the proportion of gamma-H2AX-positive cells compared to those among control cells. However, the increase in gamma-H2AX levels induced by miR-185 could not be rescued by POT1 reintroduction (Figure 2D, 2F). These results indicated that miR-185 specifically elicits telomere DNA damage by downregulating POT1 expression levels.

### MiR-185 induce telomere dysfunction via POT1-ATR signaling pathway

**Figure 3:**
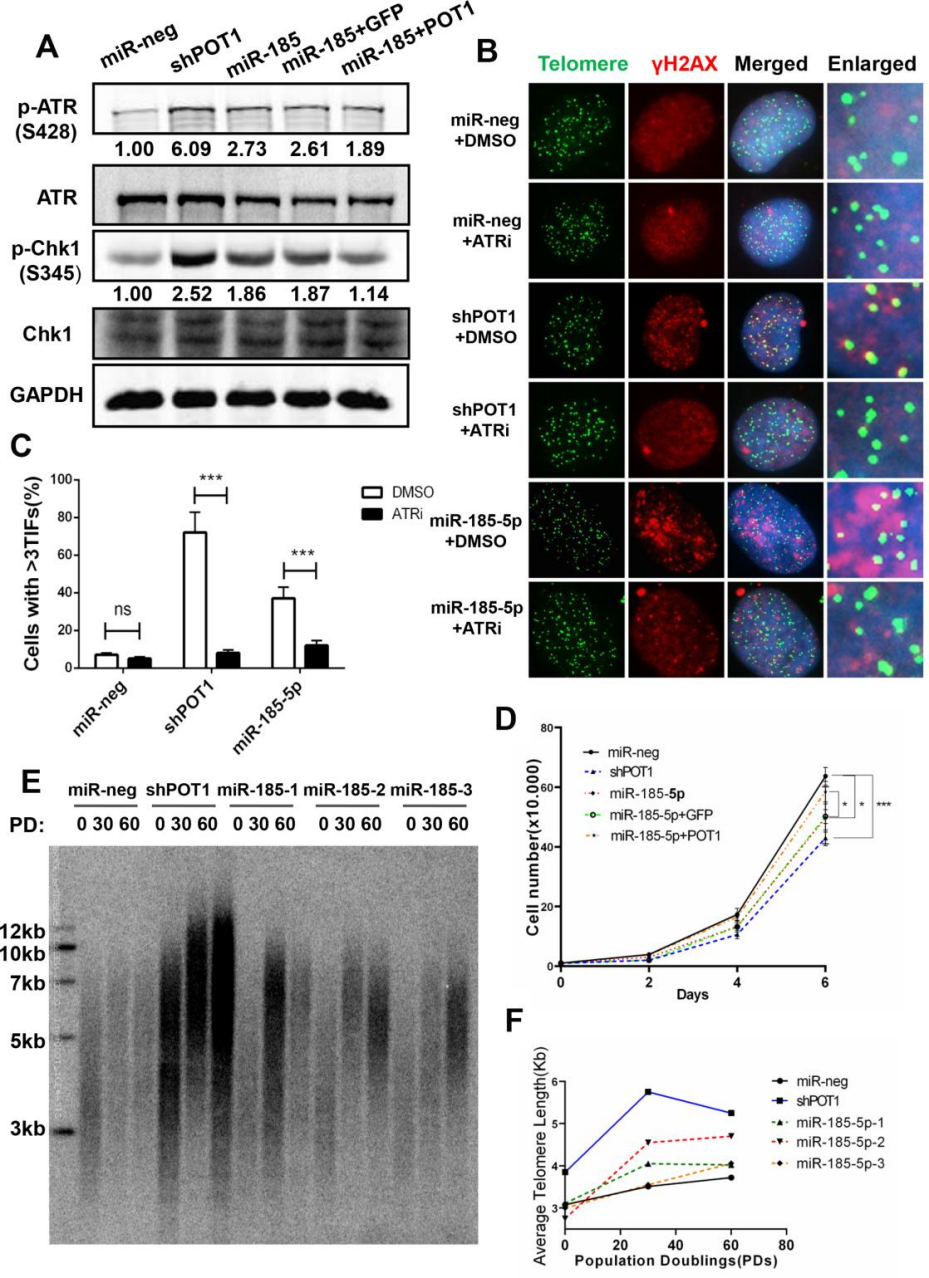
**MiR-185 increases telomere dysfunction via the ATR signaling pathway in cancer cells.** (**A**) ATR protein levels and phosphorylated Chk1 levels in POT1 knockdown, miR-185 overexpression and POT1 rescue HTC75 cells. Total Chk1 was blotted as a loading control. (**B**) After treatment with the ATR kinase inhibitor VE-821, the POT1 knockdown, miR-185 overexpression and control HTC75 cells were examined by immunostaining using anti-γ-H2AX antibody and fluorescence in situ hybridization using the TTAGGG telomere probe. (**C**) Quantification of the percentage of cells in (**B**) with more than 3 TIFs. Error bars indicate standard deviations (n=3). (**D**) Growth curve of HTC75 cells stably infected with viruses encoding empty vector (miR-Neg), shPOT1, miR-185, miR-185 plus GFP, or miR-185 plus POT1 at different time points. (**E**) Telomere restriction fragment (TRF) analysis showed the telomere length of HTC75 cells stably infected with the indicated viruses at the indicated population doublings (PD 0, PD 30, PD 60). (**F**) Quantification of telomere length in (**E**).

As the loss of POT1 activates the ATR signaling pathway and induces telomere dysfunction, we next examined whether miR-185 also induces telomere dysfunction via the ATR signaling pathway. It was reported previously that miR-185 can also target ATR and decrease its protein expression levels [28]. Consistently, the total ATR protein level reduced upon miR-185 overexpression. However, the phosphorylated ATR was upregulated upon miR-185 overexpression (Figure 3A, lane 3). Chk-1 is phosphorylated by activated ATR kinase when cells suffer single-strand DNA damage and plays a crucial role in the G2/M DNA damage checkpoint [29]. The phosphorylated Chk1 was also upregulated upon either POT1 knockdown or miR-185 overexpression, suggesting that miR-185 can activate the ATR downstream pathway (Figure 3A, lane 3). The reintroduction of POT1 abolished the increased Chk1 phosphorylation in the miR-185-overexpression group, suggesting that miR-185 activates the ATR signaling pathway via POT1 (Figure 3A, lane 5). To test whether miR-185 induces telomere dysfunction via ATR signaling, we added the ATR inhibitor VE-821 to cells with either POT1 knockdown or miR-185 overexpression. Dramatically, ATR inhibition abolished the increased TIF signals upon POT1 knockdown or miR-185 overexpression (Figure 3B, 3C). We monitored the cell growth rate and found that either POT1 knockdown or miR-185 overexpression could significantly decrease the cell proliferation rate, and reintroducing POT1 in the miR-185 overexpression group partially rescued this inhibitory effect on growth (Figure 3D). Furthermore, either POT1 knockdown or miR-185 overexpression increased the sensitivity of HTC75 cells to doxorubicin chemotherapy, which is consistent with the triggering genome instability by miR-185 or sh-POT1 (Supplementary Figure 2). Furthermore, we examined whether telomere length is also affected by miR-185. A terminal restriction fragment (TRF) assay was performed in HTC75 cells stably transfected with POT1 shRNA or miR-185 vector, and the results showed telomere lengthening in either POT1 knockdown or miR-185-overexpressing cells (Figure 3E, 3F). Taken together, these results strengthened the notion that prolonged expression of miR-185 elicits telomere DNA damage via the POT1-ATR signaling pathway and eventually lengthens telomeres.

### MiR-185 overexpression induced cellular senescence in primary cells

**Figure 4:**
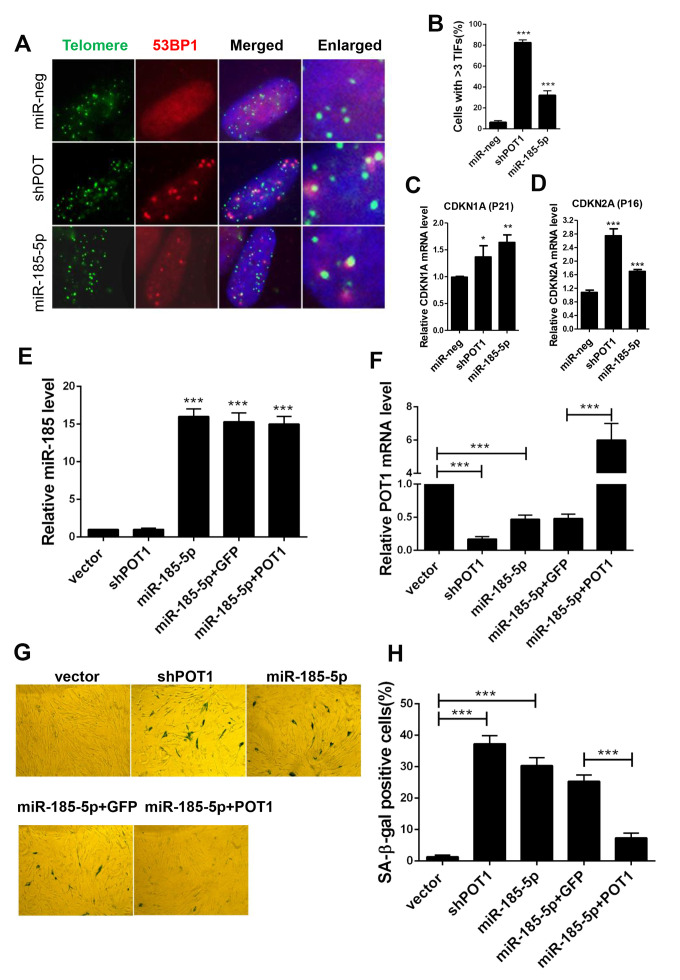
**MiR-185 overexpression induces cellular senescence in primary human fibroblast cells.** (**A**) Immunofluorescence and fluorescence in situ hybridization (IF-FISH) were performed in early passage HFF cells stably infected with viruses encoding empty vector (miR-Neg), shPOT1 or miR-185. (**B**) Quantification of the percentage of cells in (**A**) with more than 3 TIFs. (**C**) CDKN1A mRNA levels were detected by qRT-PCR. (**D**) CDKN2A mRNA level were detected by qRT-PCR. (**E**) HFF cells were stably infected with viruses encoding empty vector (miR-Neg), shPOT1, miR-185, miR-185 plus GFP, or mIR-185 plus POT1 respectively, miR-185 levels were detected by qRT-PCR. Relative levels of miR-185 were normalized using the U6 RNA. (**F**) POT1 mRNA levels were detected by qRT-PCR. Relative expression of the POT1 gene was normalized using GAPDH level. (**G**) HFF cells stably infected with viruses encoding empty vector (miR-Neg), shPOT1, miR-185, miR-185 plus GFP, or mIR-185 plus POT1 respectively, were stained for β-galactosidase activity (SA-β-gal). (**H**) Quantification of the percentage of cells in (**G**) positive for SA-β-gal staining. Error bars indicate standard deviations (n=3). P values were determined by Student’s t-test. ***P<0.001.

Since prolonged activation of the ATR signaling pathway activates P53 and elicits cell senescence in primary cells, we next examined the role of miR-185 in primary cells during replicative senescence. Human foreskin fibroblast (HFF) and MRC5 lung fibroblast cells are commonly used as replicative senescence models. Next we constructed stable lines with POT1 knockdown or miR-185 overexpression in primary HFF cells. Either POT1 knockdown or miR-185 overexpression in HFF cells induced a significant increase in TIF signals compared with those in the control cells (Figure 4A, 4B). Quantitative PCR was performed in late-passage HFF cells. Either POT1 knockdown or miR-185 overexpression in HFF cells significantly increased CDKN1A (P21) and CDKN2A (P16) expression levels (Figure 4C, 4D). Senescence-associated β-galactosidase (SA-β-gal) staining was performed to detect senescent cells. We found that either POT1 knockdown or miR-185 overexpression significantly increases the proportion of senescent cells, while the reintroduction of POT1 in miR-185-overexpressing cells abolished this phenotype (Figure 4E–4H).

**Figure 5:**
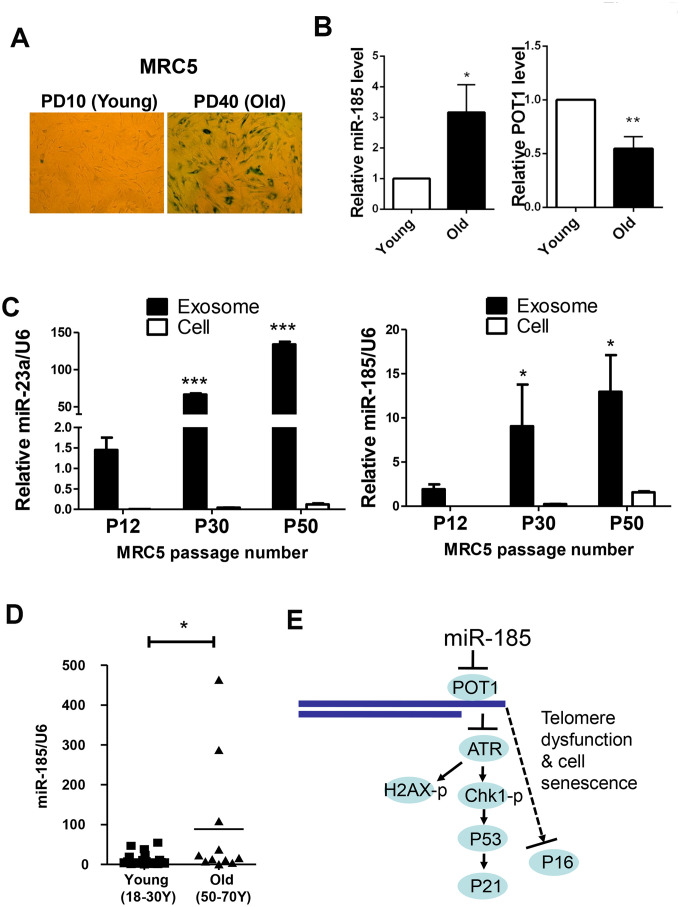
**MiR-185 represents a novel replicative senescence biomarker *in vitro* and *in vivo*.** (**A**) Young wild-type MRC5 cells were passaged to the old (From PD10 to PD40) and were stained for β-galactosidase activity respectively. (**B**) The relative expression levels of miR-185 and POT1 in cells from (**A**) were detected and normalized using U6 and GAPDH levels respectively. (**C**) MRC5 cells from different passages were cultured, and exosomes were obtained by ultracentrifugation. The relative expression levels of miR-23a and miR-185 were detected and normalized using U6. (**D**) The relative expression levels of miR-185 from young (males aged 18-30) and old (males aged 50-70) people were detected and normalized using U6 (n=19 and 11 respectively). P values were determined by Student’s t-test. *P<0.05. (**E**) Proposed model by which miR-185 induces telomere dysfunction and cell senescence via the POT1 pathway.

We also detected the levels of miR-185 and POT1 during cell replicative senescence. Consistent with the anti-aging function of POT1, POT1 levels dramatically decreased during replicative senescence. Interestingly, the miR-185 levels were significantly increased in senescent MRC5 cells compared to young cells, suggesting that miR-185 and POT1 are negatively correlated during the replicative senescence process (Figure 5A, 5B). The antibiotic Zeocin can also induce senescence in HFF and MRC5 cells (Supplementary Figure 3A). Consistently, miR-185 expression was significantly increased after Zeocin treatment, while POT1 mRNA expression was decreased after Zeocin treatment; In both HFF and MRC5 cells, ATR mRNA levels did not change significantly (Supplementary Figure 3B, 3C). These results indicate a negative correlation between miR-185 and POT1 levels in both replicative and induced senescence. Since miRNAs can be detected in extracellular spaces such as serum or urine and exosomes may mediate cell-cell communication during the aging process, we next isolated exosomes from MRC5 cells at different passages. MiR-23a was a previously identified SA-miRNA significantly increased during the senescence process. We first detected miR-23a and found a significant enrichment and increase of miR-23a in exosomes from older passaged MRC5 cells compared to younger cells (P50 vs. P30 and P12), which is consistent with our previous report [23]. Strikingly, miR-185 levels were also significantly increased in exosomes from older passaged MRC5 cells compared to younger cells (Figure 5C). Furthermore, we isolated miRNAs from the sera of humans of different ages (18 y-70 y). The serum miR-185 levels in old males aged 50-70 y were significantly higher than those in young males aged 18-30 y, suggesting that serum miR-185 may be a biomarker of human aging (Figure 5D). Taken together, these results confirmed that miR-185 promotes senescence by downregulating POT1 expression to and deprotecting telomeres.

## DISCUSSION

In this study, a combination of bioinformatics tools and dual-luciferase reporter assay mini-screening was used to identify miRNAs that can target the telomere protection protein POT1. The use of an integrated bioinformatic prediction tool avoided large-scale screening, which is time-consuming and costly. Most of the predicted miRNA candidates (11 out of 12) showed significant decreased relative luciferase activity, suggesting that the prediction tool was reliable. Additionally, the corresponding miR-185 binding region was confirmed by generating a mutant POT1 3’-UTR reporter. Overexpression miR-185 increased telomere dysfunction-induced foci signals in both cancer cells and primary human diploid cells. Moreover, miR-185 accelerated the senescence process of primary human cells in a POT1-dependent manner. A possible model is proposed in Figure 5E. Collectively, our results identified a new senescence-associated miRNA and provided a new mechanism by which telomere function is modulated.

POT1 is a key protein that safeguards genome stability. POT1 mutation was found in several cancers [30]. By using animal models, a POT1 mutation was reported to promote genome instability and fuel tumorigenesis [15, 31]. Aging is a well-known antitumor mechanism but also promotes tumorigenesis. Enhanced of genome instability during senescence contributes greatly to tumorigenesis. In this study, we identified miR-185 as a novel pro-senescence miRNA in human serum. Interestingly, miR-185 is a known tumor suppressor in several cancer types, including melanoma, renal cell carcinoma and breast cancer, and miR-185 was reported to increase the radiation induced proliferation inhibition in different cancer types [32–35]. Several studies also indicate low miR-185 expression correlates with worse cancer patient prognosis [32, 36, 37]. In our study, either POT1 knockdown or miR-185 overexpression in established cancer cells significantly decreased cell proliferation (Figure 3D). Consistent with our findings, the expression of miR-185 was shown to be negatively correlated with POT1 in some kinds of cancers, such as breast cancer, colon and rectal adenocarcinoma, and cutaneous melanoma (http://starbase.sysu.edu.cn/targetSite.php, Pan-Cancer miRNA-Target Pearson Correlation Analysis, r=-0.21796, -0.24041 and -0.10678, respectively, with P<0.05). These results support our finding that POT1 might be downregulated by miR-185 in the aging and tumorigenesis process.

Surprisingly, we found that miR-185 overexpression increased the overall genome-wide DNA damage signal in HTC75 cells (Figure 2F). However, the increased phosphorylation of H2AX caused by miR-185 overexpression could not be rescued by POT1, suggesting that miR-185 also influences genome stability via other pathways. Phosphorylation of H2AX relies on ATM, DNA-PKcs or ATR, depending on the context. Interestingly, ATR was also found to be a miR-185-targeted protein. Mouse with ATR. depletion exhibited a premature phenotype [38]. In our work, although the ATR level was slightly decreased upon miR-185 overexpression, phosphorylation levels of Chk1, which is downstream of ATR, were still elevated, suggesting that the ATR-Chk1 signaling pathway was activated. Additionally, the pro-aging phenotype caused by miR-185 overexpression was rescued by the reintroduction of POT1, suggesting that miR-185 specifically promotes cell senescence via POT1. Previously, defects in the ATR gene were found to cause Seckel syndrome 1, a premature aging disease [39, 40]. Consistent with our results, activation of ATR was found to cause cell cycle arrest as well as P53-dependent cellular senescence [41]. We found that miR-185 was significantly increased in senescent cells, similar to miR-23a, a previously identified aging-related miRNA. Interestingly, miR-185 was recently found to be decreased in the sera and placentas of patients with gestational diabetes mellitus [42]. MiR-185 in human serum might be an aging marker and the recycling of miR-185 may play important roles in cell-cell contact to transduce pro-aging signaling. In summary, miR-185 may be a potential target in therapeutic application of the anti-aging field.

## MATERIALS AND METHODS

### Vectors and compounds

The human miRNA expression vectors mentioned in this paper were constructed following previously described protocol [27]. MiR-neg, the miRNA negative control vector was obtained from our previous study [23]. The human POT1 3’-UTR was amplified by PCR and subsequently cloned into the downstream of the Renilla Luciferase reporter in the psiCHECK-2 vector (Promega, Madison, WI, USA). Mutation of the seed region of miR-185 in POT1 3’-UTR was achieved by quick-change PCR using the POT1 3’-UTR vector as a template. The POT1-shRNA sequence (shPOT1: 5’- GTACTAGAAGCCTATCTCACT-3’) was cloned into the lentiviral vector pLKO.1-puro. GFP and full-length human POT1 cDNA were cloned into a modified pBabe-based lentiviral vectors with C-terminus SFB tag (SFB tag includes S tag, Flag epitope tag, and Streptavidin-binding peptide tag). ATR inhibitor VE-821 (Sigma-Aldrich, SML1415) was used at a final concentration of 500 nM. Doxorubicin hydrochloride (Santa Cruz Biotechnology, sc-200923) was stock in 100 μM and used at indicated concentration.

### Cell lines

The HTC75, HEK293T and MRC5 cell lines were purchased from the American Type Culture Collection. Cells were cultured in DMEM with 4.5 g/L glucose, L-glutamine and sodium pyruvate, supplemented with 10% fetal bovine serum and 100 U/mL penicillin/ streptomycin. Lentivirus was generated from HEK 293T. Stable cell lines were constructed by two rounds of lentiviral infection at a 24-hour interval and with subsequent puromycin (1 μg/mL) selection for 3 days. To generate the stable rescue cell lines, stable miR-185 overexpression cells were infected again using lentiviruses encoding GFP-SFB or POT1-SFB.

### Dual-luciferase reporter assay

A dual-luciferase reporter assay was carried out according to the manufacturer’s instructions (Promega, E1960). Briefly, HEK293T cells were plated into a 24-well dish and cultured for 24 hours prior to cotransfection. The POT1 3’-UTR (150 ng) and individual miRNA expression vectors (450 ng) or miRNA negative control vector were cotransfected into the prepared HEK293T cells using PEI (polyethylenimine, 1 mg/ml, Polysciences, 24765-1). Cells were harvested after 48 hours post-transfection and lysed with 1×passive buffer. Firefly and Renilla luciferase activities were measured with a Dual-Luciferase Reporter Assay Kit (Promega) according to the manufacturer’s protocol. The ratio of Renilla luciferase activity to Firefly luciferase activity in cells transfected with individual miRNA expression vectors was normalized to that in cells transfected with the miRNA negative control vector. Lysis in each cotransfection experiment was measured in triplicate. Three independent cotransfection experiments were performed. The mean values and the standard deviations were determined by three independent cotransfection experiments. Significant differences between cells transfected with each individual miRNA vector and the miRNA negative control vector were analyzed by Student’s t-test.

### Western blotting and antibodies

Whole-cell lysates were prepared as follows: cells were harvested, resuspended in commercial RIPA buffer (Beyotime, P0013B), and then subjected to sonication followed by centrifugation. Western blotting was carried out as previously described [14]. The primary antibodies used for western blotting were as follows: mouse monoclonal anti-GAPDH (Abmart, 3B3), rabbit polyclonal anti-POT1 (Novus, NB500-176), goat polyclonal anti-ATR (Santa Cruz Biotechnology, sc-1887), rabbit polyclonal anti-phospho-ATR (Abcam, ab178407), mouse monoclonal anti-Chk1 (Santa Cruz Biotechnology, sc-377231), rabbit monoclonal anti-phospho-Chk1 (Cell Signaling Technology, No.2348L). The secondary antibodies used were HRP-conjugated donkey-anti-goat (Santa Cruz Biotechnology, sc2020), fluorescein-conjugated IRDye-680CW goat anti-mouse (LI-COR, 926-32220) and IRDye-800CW goat anti-rabbit (LI-COR, 926-32211).

### Immunofluorescence (IF) and Fluorescence-in situ hybridization (IF-FISH)

Immunofluorescence was conducted as previously described [14]. For IF experiments, cells were seeded on glass coverslips, fixed with 4% paraformaldehyde, permeabilized with 0.5% Triton X-100, blocked with 5% goat serum prior to primary and secondary antibody incubation, and finally subjected to DAPI (Vector Laboratories, H-1200) staining. The following primary antibodies were used in this experiment: rabbit monoclonal anti-phospho-histone-H2AX (Cell Signaling Technology, 9718S); and rabbit polyclonal anti-53BP1 (Novus, NB100-304). The secondary antibody was Alexa Fluor 555-conjugated donkey anti-rabbit (Invitrogen, A-31572). Fluorescence microscopy was carried out with an ImagerZ1 microscope (Carl Zeiss, Germany) and images obtained were processed with AxioVision LE software. TIFs in at least 300 cells were quantified.

For IF-FISH experiments, hybridization with the PNA-TelG-Cy3 probe (Panagene, F1006-5) for 2 hours at 37 °C was followed by immunofluorescence after secondary antibody incubation.

### Real-time quantitative RT-PCR (qRT-PCR)

RNA was isolated from the cells with TRIzol (Thermo Fisher Scientific, 15596026), after which residual genomic DNA was removed with DNaseI (Invitrogen, 1827704). For mRNA detection, a RevertAid First Strand cDNA Synthesis Kit (Thermo Fisher Scientific, #K1622) was applied for reverse transcription to convert total mRNA to first-strand cDNA. The human serum collection was approved by the Ethics Committee. Human sera were obtained from 18- to 70-year-old healthy males and frozen at -80 °C. MiRNAs were extracted from 1 mL each of human sera from individuals of different ages using the miRNeasy Serum/Plasma Kit (QIAGEN, 217184). For miRNA detection, reverse transcription was carried out with ReverTra Ace α Transcriptase (TOYOBO, FSK-100B). Real-time qPCR was performed on an ABI OneStep Plus system (Applied Biosystems) using the Powerup SYBR Green Master Mix (Applied Biosystems, A25742). The qRT-PCR primers for mRNA detection were as follows: GAPDH-forward primer (5’-GGAGCGAGATCCCTCCAAAAT-3’), GAPDH-reverse primer (5’-GGCTGTTGTCATACTTCTCATGG-3’), POT1-forward primer (5′-GAAGTGGACGGAGCATCATT-3′), POT1-reverse primer (5′-TTTGTAGCCGATGGATGTGA-3′), ATR-forward primer (5’-TGCAGTAATGTCAATGGTTGG-3’), and ATR-reverse primer (5’-CTGGAACTTCAAAGGTTTCTCC-3’).

The following qRT-PCR primers used for miRNA detection were purchased from RiboBio (Guangzhou, China): miR-185-reverse transcription primer (RiboBio, ssD1210318482), miR-185-forward primer (RiboBio, ssD1210318481), miR-185-reverse primer (RiboBio, ssD089261711), U6-reverse transcription primer (RiboBio, ssD0904071008), U6-forward primer (RiboBio, ssD0904071006), U6-reverse primer (RiboBio, ssD0904071007)

### SA-β-gal assay and cell growth curves

Briefly, 1×10^4^ cells were plated into 6-well plates at day 7 post-selection. The activity of senescence-associated β-galactosidase (SA-β-gal) in different stable primary cells was detected by Lysosomal β-Galactosidase Staining Kit (Beyotime, C0605) according to the manufacture’s instruction. At least 300 cells were analyzed for the SA-β-gal positive scoring.

## Supplementary Material

Supplementary Figures

Supplementary Table 1
