# Supplementary material for: MiR-185 targets POT1 to induce telomere dysfunction and cellular senescence
Source: Aging (Albany NY). 2020 Jul 18;12(14):14791–807. doi: 10.18632/aging.103541 (PMC7425516; doi:10.18632/aging.103541)
Supplement: Supplementary Figures [file aging-12-103541-s002..pdf]

SUPPLEMENTARY FIGURES

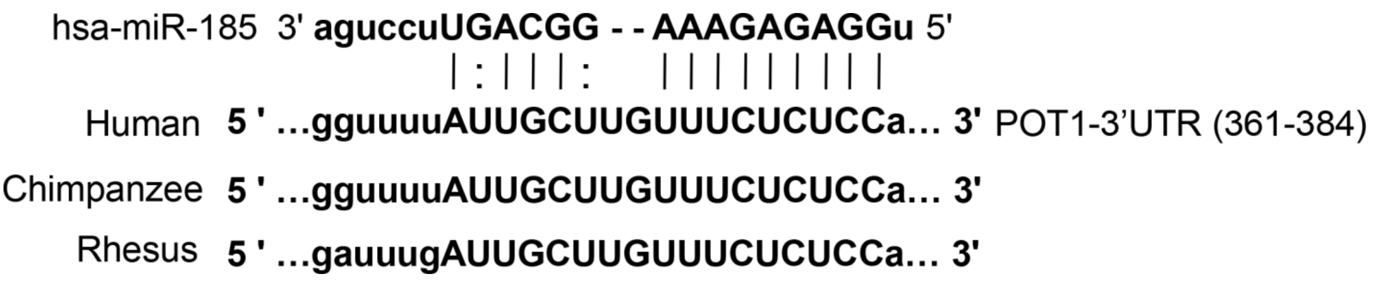

Supplementary Figure 1. The predicted target site of miR-185 within POT1 3’UTR appears conserved across primate species.

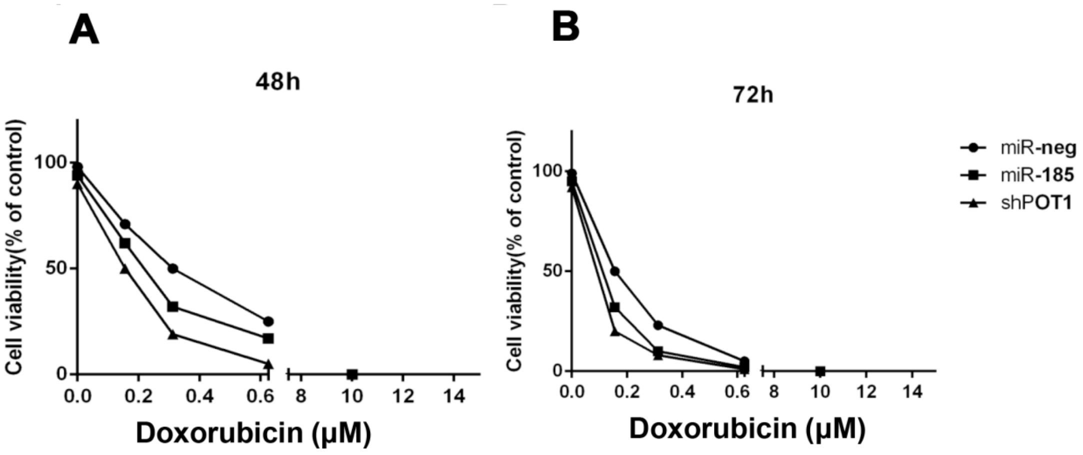

Supplementary Figure 2. The sensitivity to chemotherapy drug doxorubicin of HTC75 cells after 48 hours (**A**) and 72 hours (**B**) with POT1 knocking down and miR185 overexpression.

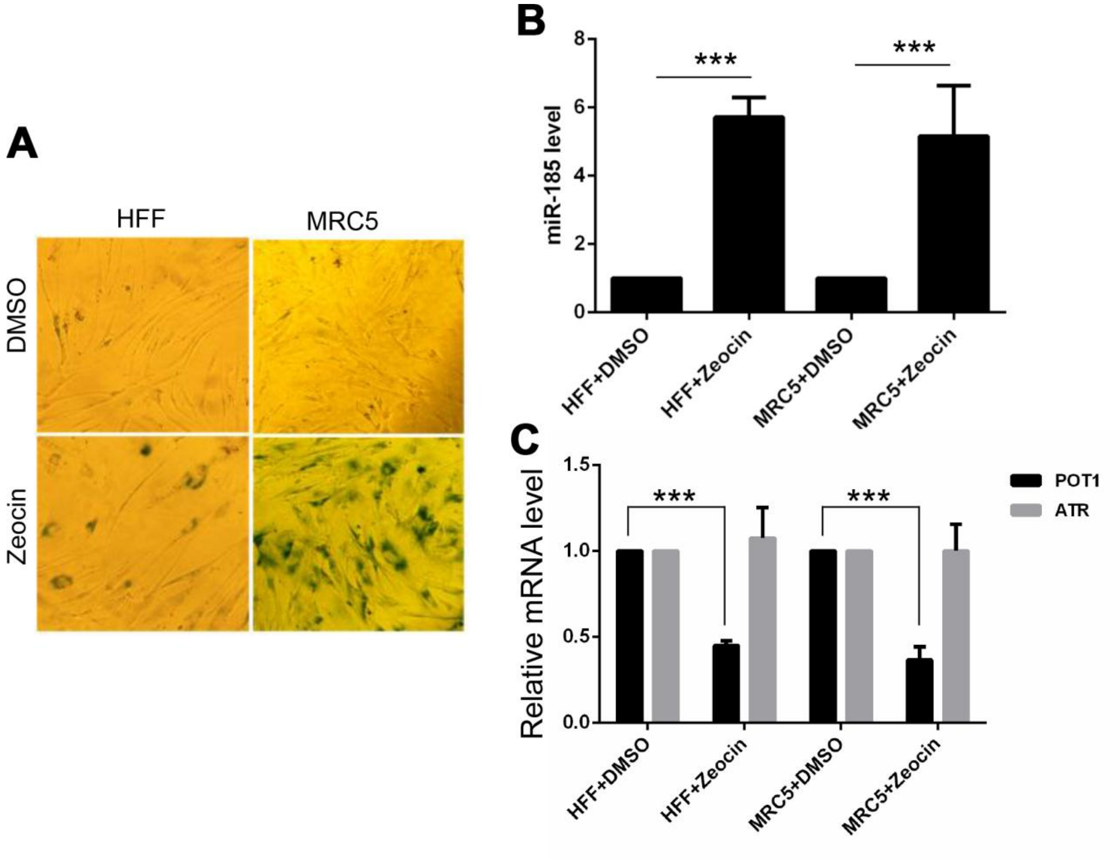

Supplementary Figure 3. miR-185 and POT1 may negatively correlated during the Zeocin induced senescent process in primary cells. (**A**) Early passage HFF and MRC5 cells that were treated by Zeocin were stained for b-galactosidase activity. (**B**, **C**) The relative expression of miR-185 (**B**), POT1 and ATR from cells of inducd senescence (**C**) were detected and normalized using U6 and GAPDH level respectively.
