# Supplementary material for: MiR-185 targets POT1 to induce telomere dysfunction and cellular senescence
Source: Aging (Albany NY). 2020 Jul 18;12(14):14791–807. doi: 10.18632/aging.103541 (PMC7425516; doi:10.18632/aging.103541)
Supplement: Supplementary Table 1 [file aging-12-103541-s001..pdf]

SUPPLEMENTARY TABLE

Supplementary Table 1. miRNAs predicted to target POT1 by starBase v2.0.

Name	Gene	Position	targetScanSites	picTarSites	RNA22Sites	PITASites	miRandaSites *
hsa-miR-340-5p	POT1	chr7:124463403-124463424[-]	0[0]	0[0]	0[0]	0[0]	1[1]
hsa-miR-99b-5p	POT1	chr7:124463855-124463878[-]	0[0]	0[0]	0[0]	0[0]	247[1]
hsa-miR-224-5p	POT1	chr7:124463562-124463582[-]	0[0]	0[0]	0[0]	0[0]	5[1]
hsa-miR-539-5p	POT1	chr7:124463613-124463635[-]	0[0]	0[0]	0[0]	0[0]	20[3]
hsa-miR-382-5p	POT1	chr7:124463510-124463533[-]	0[0]	0[0]	0[0]	0[0]	39[1]
hsa-miR-410-3p	POT1	chr7:124463402-124463422[-]	0[0]	0[0]	0[0]	0[0]	1[1]
hsa-miR-543	POT1	chr7:124463570-124463591[-]	0[0]	0[0]	0[0]	0[0]	3[1]
hsa-miR-1271-5p	POT1	chr7:124463368-124463389[-]	0[0]	0[0]	0[0]	0[0]	8[1]
hsa-miR-433-3p	POT1	chr7:124463430-124463437[-]	1[1]	0[0]	0[0]	0[0]	1[1]
hsa-miR-21-5p	POT1	chr7:124463751-124463773[-]	0[0]	0[0]	0[0]	0[0]	74[1]
hsa-miR-185-5p	POT1	chr7:124463632-124463655[-]	0[0]	0[0]	0[0]	0[0]	17[2]
hsa-miR-216b-5p	POT1	chr7:124463617-124463638[-]	0[0]	0[0]	0[0]	0[0]	20[3]
hsa-miR-9-5p	POT1						
	chr7:124463366-124463388[-]	0[0]	0[0]	0[0]	0[0]	8[1]	
hsa-miR-182-5p	POT1	chr7:124463368-124463389[-]	0[0]	0[0]	0[0]	0[0]	8[1]
hsa-miR-96-5p	POT1	chr7:124463368-124463391[-]	0[0]	0[0]	0[0]	0[0]	9[2]
hsa-miR-383-5p	POT1	chr7:124463756-124463777[-]	0[0]	0[0]	0[0]	0[0]	74[1]

*Target Information from miRanda Program: ClipSeq ReadNum [ClipSeq peakCluster].
Four miRNAs in grey, hsa-miR-9-5p, hsa-miR-182-5p, hsa-miR-96-5p and hsa-miR-383-5p not included in our established miRNA expression library (Zhou et al., 2013), were not performed experiments further.
